# Do Interventions that Promote Awareness of Rights Increase Use of Maternity Care Services? A Systematic Review

**DOI:** 10.1371/journal.pone.0138116

**Published:** 2015-10-07

**Authors:** Asha S. George, Casey Branchini, Anayda Portela

**Affiliations:** 1 Health Systems Program, Department of International Health, Johns Hopkins School of Public Health, Baltimore, Maryland, United States of America; 2 Maternal, Newborn, Child and Adolescent Health, World Health Organization, Geneva, Switzerland; The Hospital for Sick Children, CANADA

## Abstract

Twenty years after the rights of women to go through pregnancy and childbirth safely were recognized by governments, we assessed the effects of interventions that promote awareness of these rights to increase use of maternity care services. Using inclusion and exclusion criteria defined in a peer-reviewed protocol, we searched published and grey literature from one database of studies on maternal health, two search engines, an internet search and contact with experts. From the 707 unique documents found, 219 made reference to rights, with 22 detailing interventions promoting awareness of rights for maternal and newborn health. Only four of these evaluated effects on health outcomes. While all four interventions promoted awareness of rights, they did so in different ways. Interventions included highly-scripted dissemination meetings with educational materials and other visual aids, participatory approaches that combined raising awareness of rights with improving accountability of services, and broader multi-stakeholder efforts to improve maternal health. Study quality ranged from weak to strong. Measured health outcomes included increased antenatal care and facility birth. Improvements in human rights outcomes such as availability, acceptability, accessibility, quality of care, as well as the capacity of rights holders and duty bearers were also reported to varying extents. Very little information on costs and almost no information on harms or risks were described. Despite searching multiple sources of information, while some studies did report on activities to raise awareness of rights, few detailed how they did so and very few measured effects on health outcomes. Promoting awareness of rights is one element of increasing demand for and use of quality maternity care services for women during pregnancy, birth and after birth. To date efforts have not been well documented in the literature and the program theories, processes and costs, let alone health effects have not been well evaluated.

## Introduction

### Rationale

The rights of women to go through pregnancy and childbirth safely was first agreed to by countries in 1994 through the Program of Action for the International Conference on Population and Development [1]. It was reiterated in the ten year review of the Safe Motherhood Initiative [2], reaffirmed by United Nations agencies [3, 4] and endorsed by the Secretary General in his Global Strategy on Women and Children [5] and its corresponding UN Commission on Information and Accountability (COIA). Most recently, the Office of the United Nations High Commissioners for Human Rights (OHCHR) linked maternal mortality with human rights [6], issued guidance on the issue [7] and subsequently adopted a resolution whereby member states report progress to the Human Rights Council [8].

OHCHR [7] states:

Human rights are about empowerment and entitlement of people with respect to certain aspects of their lives, including their sexual and reproductive health. International human rights law includes fundamental commitments of States to enable women to survive pregnancy and childbirth as part of their enjoyment of sexual and reproductive health rights and living a life of dignity. Sound public health practice is crucial to enable States to fulfil these basic rights, but it must be complemented by broader measures to address women’s empowerment.

**Fig 1:**
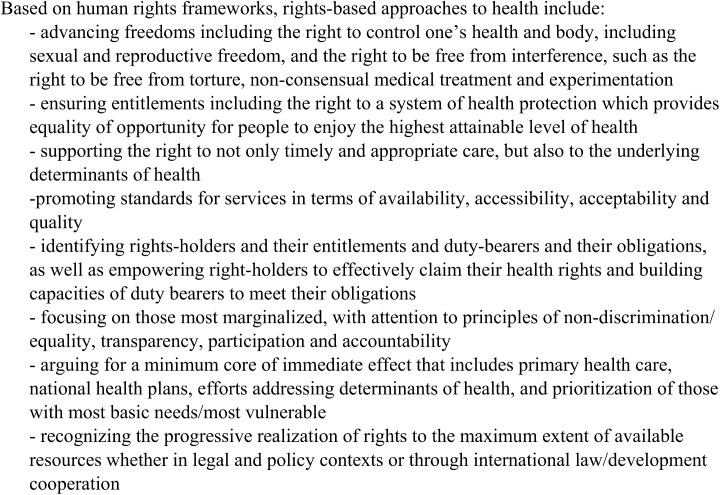
Rights-based approaches to health.

The guidance provided by OHCHR is welcome, as health professionals and programs do struggle to operationalize rights-based approaches to health [9] derived from human rights frameworks and agreements [3, 10–12] (Fig 1). Maternal and newborn health programs endeavor to embrace such an approach through a range of efforts involving information and awareness raising; community participation; service delivery accountability; and support for gender equality and women’s rights, preferences and needs. Most recently, attention to the humanization of birth, and/or disrespect and abuse during childbirth re-emphasizes the importance of human rights for maternal health, with WHO and partners recommending greater support for research and action with emphasis on quality of care; explicit reference to rights; capacity building and accountability within health systems; and engagement with diverse stakeholders [13].

Despite the need to engage public health practitioners and support implementation strategies, reviews have not systematically assessed interventions or implementation strategies to operationalize human rights approaches, with the exception of Ferguson and Halliday [14], who examined how human rights approaches were implemented through community participation. Previous reviews articulate the relevance and importance of rights-based approaches for maternal and newborn health [15] [16] [17] [18], but have not assessed how efforts to operationalize rights programmatically have impacted the use of maternity care services to improve maternal and newborn health.

### Objectives

In this review, we systematically assessed the evidence available on interventions that promote awareness of rights to increase use of maternity care services. We asked the question: What interventions to promote awareness of human rights/sexual and reproductive rights /right to access to quality care are effective in increasing demand for and use of health care to improve maternal and newborn health outcomes? Inclusion and exclusion criteria were developed to find studies across all populations with participants defined as pregnant women or women in labor and with interventions aimed to improve awareness of rights among women, men, community members or health workers and program administrators, compared against instances where such awareness was not promoted. Critical outcomes included birth with a skilled attendant or in a facility, use of antenatal care, use of postnatal care for the infant and mothers, as well as satisfaction with the birth experience. Important outcomes were those related to health and rights awareness. All study designs were included provided that they reported on desired outcomes, with study outcomes compared with control groups or with data collection prior and post the intervention. Studies that only described an intervention but did not measure any health outcomes were not included for this analysis.

## Methods

### Protocol and registration

A protocol was developed and reviewed by WHO and peers before being finalized and implemented.

### Information sources and search

We identified references through a data base developed through a systematic mapping of maternal health studies and systematic reviews focused on health system and community-based interventions for improving maternal health and for reducing maternal health inequities in low and middle-income countries (LMICs) (http://eppi.ioe.ac.uk/webdatabases4/Intro.aspx?ID=11). The data base was completed as part of two research projects: the Multilateral Association for Studying health inequalities and enhancing north-south and south-south COoperaTion project (MASCOT), which received funding from the European Union’s Seventh Framework Programme FP7/2007-2013 under grant agreement number 282507; and the Maternal Health in South Africa and Rwanda (MH-SAR) project, funded by the Netherlands Organization for Scientific Research (NWO/WOTRO). The ***MASCOT/MH-SAR*** mapping includes references for 2000–2012 for low- and middle-income countries (LMICs) in the following languages: Arabic, English, French, Japanese, Portuguese and Spanish. A team supported by WHO coded articles related to health promotion interventions for maternal and newborn health including for health education, promotion of human rights, social accountability, and respectful care.

In addition, ***PubMed*** was queried using the key concepts from the study’s research question: (1) Human Rights; (2) Maternal/Newborn/Child Health; and (3) LMICs (Table 1). Specified search terms identified for each concept were linked using the Boolean “AND” operator. We searched for items published in Arabic, English, French, Japanese, Portuguese and Spanish language publications published between 2010 and December 31, 2014. A publication alert was set up to identify articles meeting these criteria from January 1, 2014 and May 31, 2014.

**Table 1:** Concepts and terms for PubMed search: January 1, 2010—May 31, 2014.

*Concepts*	*Search terms*
**Human Rights**	"Human Rights"[tw] OR "Rights-based"[tw] OR "Basic-rights"[tw] OR "Patient-charter"[tw] OR "Civil rights"[tw] OR "Legal rights"[tw] OR "Patient rights"[tw] OR "Reproductive rights"[tw] OR "Social justice"[tw] OR "Women’s rights"[tw] OR "Informed consent"[tw] OR "Personal autonomy"[tw] OR "Confidentiality"[tw]
**Maternal and Child Health**	"maternal health"[tw] OR "child health" OR "newborn health" OR "Obstetric care"[tw] OR "Perinatal care"[tw] OR "Maternal health services"[tw] OR "Postnatal care"[tw] OR "Prenatal care"[tw] OR "Maternal mortality"[tw] OR "Delivery obstetric"[tw] OR "Pregnancy"[tw]
**Low-income setting**	*Refers to low- or middle- income countries or low-income settings in high-income countries*. *In addition to the search terms listed below*, *the name of each low- and middle-income (LMICs) country was also included as per the classification of the World Bank*. "developing country"[tiab] OR "developing countries"[tiab] OR "developing nation"[tiab] OR "developing nations"[tiab] OR "developing population"[tiab] OR "developing populations"[tiab] OR "developing world"[tiab] OR "less developed country"[tiab] OR "less developed countries"[tiab] OR "less developed nation"[tiab] OR "less developed nations"[tiab] OR "less developed population"[tiab] OR "less developed populations"[tiab] OR "less developed world"[tiab] OR "lesser developed country"[tiab] OR "lesser developed countries"[tiab] OR "lesser developed nation"[tiab] OR "lesser developed nations"[tiab] OR "lesser developed population"[tiab] OR "lesser developed populations"[tiab] OR "lesser developed world"[tiab] OR "under developed country"[tiab] OR "under developed countries"[tiab] OR "under developed nation"[tiab] OR "under developed nations"[tiab] OR "under developed population"[tiab] OR "under developed populations"[tiab] OR "under developed world"[tiab] OR "underdeveloped country"[tiab] OR "underdeveloped countries"[tiab] OR "underdeveloped nation"[tiab] OR "underdeveloped nations"[tiab] OR "underdeveloped population"[tiab] OR "underdeveloped populations"[tiab] OR "underdeveloped world"[tiab] OR "middle income country"[tiab] OR "middle income countries"[tiab] OR "middle income nation"[tiab] OR "middle income nations"[tiab] OR "middle income population"[tiab] OR "middle income populations"[tiab] OR "low income country"[tiab] OR "low income countries"[tiab] OR "low income nation"[tiab] OR "low income nations"[tiab] OR "low income population"[tiab] OR "low income populations"[tiab] OR "lower income country"[tiab] OR "lower income countries"[tiab] OR "lower income nation"[tiab] OR "lower income nations"[tiab] OR "lower income population"[tiab] OR "lower income populations"[tiab] OR "underserved country"[tiab] OR "underserved countries"[tiab] OR "underserved nation"[tiab] OR "underserved nations"[tiab] OR "underserved population"[tiab] OR "underserved populations"[tiab] OR "underserved world"[tiab] OR "under served country"[tiab] OR "under served countries"[tiab] OR "under served nation"[tiab] OR "under served nations"[tiab] OR "under served population"[tiab] OR "under served populations"[tiab] OR "under served world"[tiab] OR "deprived country"[tiab] OR "deprived countries"[tiab] OR "deprived nation"[tiab] OR "deprived nations"[tiab] OR "deprived population"[tiab] OR "deprived populations"[tiab] OR "deprived"[tiab] OR "poor country"[tiab] OR "poor countries"[tiab] OR "poor nation"[tiab] OR "poor nations"[tiab] OR "poor population"[tiab] OR "poor populations"[tiab] OR "poor world"[tiab] OR "poorer country"[tiab] OR "poorer countries"[tiab] OR "poorer nation"[tiab] OR "poorer nations"[tiab] OR "poorer population"[tiab] OR "poorer populations"[tiab] OR "poorer world"[tiab] OR "developing economy"[tiab] OR "developing economies"[tiab] OR "less developed economy"[tiab] OR "less developed economies"[tiab] OR "lesser developed economy"[tiab] OR "lesser developed economies"[tiab] OR "under developed economy"[tiab] OR "under developed economies"[tiab] OR "underdeveloped economy"[tiab] OR "underdeveloped economies"[tiab] OR "middle income economy"[tiab] OR "middle income economies"[tiab] OR "low income economy"[tiab] OR "low income economies"[tiab] OR "lower income economy"[tiab] OR "lower income economies"[tiab] OR "low gdp"[tiab] OR"low gnp"[tiab] OR "low gross domestic"[tiab] OR "low gross national"[tiab] OR "lower gdp"[tiab] OR"lower gnp"[tiab] OR"lower gross domestic"[tiab] OR "lower gross national"[tiab] OR lmic[tiab] OR lmics[tiab] OR "third world"[tiab] OR "lami country"[tiab] OR "lami countries"[tiab] OR "transitional country"[tiab] OR "transitional countries"[tiab]

A ***general Internet search with Google/Google Scholar*** was undertaken to assist in the identification of additional studies including grey literature. Free text searching was implemented using the key concepts related to the research question (i.e. human rights, maternal/newborn/child health and low-income setting). Language of publication remained limited to the filters applied in the MASCOT/MH-SAR database search (Arabic, English, French, Japanese, Portuguese and Spanish).

The Internet search for relevant articles and reports was iterative. Along with pre-existing knowledge, the authors first identified and scanned ***four “Gateway” Internet sites*** (Table 2) to identify relevant documents (toolkits, project reports, journal articles, etc.). If a website contained a searchable database, the authors queried it for both peer-reviewed and grey literature using the concepts applied in the PubMed search (Human Rights; Maternal, Neonatal and Child Health; and Low-income). From these Gateway sites, we scanned links to all other websites for any potentially relevant organizations, researchers, or projects related to maternal health and human rights in a low-income country or setting.

**Table 2:** Internet "Gateway Sites" and Linked URLs.

*#*	***Internet Gateway Sites***	***URL for Internet Gateway Sites***
1	USAID’s Translating Research into Action (TRAction) Project	**http://www.tractionproject.org/research-areas/access-skilled-care**
2	Engender Health’s Maternal Health Task Force	**http://www.genderhealth.org/the_issues/maternal_health/**
3	International Initiative on Maternal Mortality and Human Rights (IIMMHR)	**http://righttomaternalhealth.org/resource/hr-based-approaches**
4	The White Ribbon Alliance	**http://whiteribbonalliance.org/**
*#*	***Organization/Project Linked to Gateway Site***	***URL Linked to Gateway Site***
**1**	Amnesty International	http://www.amnestyusa.org/
**2**	Asian-Pacific Resource & Research Centre for Women (ARROW)	http://www.arrow.org.my
**3**	Association of Reproductive Health Professionals	https://www.arhp.org
**4**	Averting Maternal Death and Disability (AWMDD)	http://www.amddprogram.org
**5**	CARE International	http://www.care-international.org/what-we-do/maternal-health.aspx
**6**	Center for Reproductive Rights (CCR)	http://reproductiverights.org/en
**7**	Danish Ministry of Foreign Affairs	http://um.dk/en
**8**	EQUINET	http://www.equinetafrica.org
**9**	Family Care International (FCI)	http://www.familycareintl.org/en/home
**10**	Health Equity Group (HEG)	http://righttomaternalhealth.org/node/42
**11**	Interagency Gender Working Group (IGWG), USAID	http://www.igwg.org/Publications
**12**	International Budget Project	http://internationalbudget.org
**13**	International Planned Parenthood Foundation (IPFF)	http://www.ippf.org
**14**	International Women’s Initiative	http://www.internationalwomensinitiative.or
**15**	Kvinna till Kvinna	http://kvinnatillkvinna.se/en/
**16**	Linangan ng Kababaihan (Likhaan Center for Women's Health)	http://www.amddprogram.org/
**17**	Maternal Health Task Force (MHTF)	http://www.mhtf.org
**18**	Office of the UN High Commissioner for Human Rights	http://www.ohchr.org
**19**	Physicians for Human Rights (PHR)	http://physiciansforhumanrights.org
**20**	Reproductive Rights Alliance Malaysia	http://www.rraam.org
**21**	SAYAHOG	http://www.sahayogindia.org
**22**	University of Essex, Human Rights Centre	http://www.essex.ac.uk/hrc/

Finally, direct contact was also made with experts (n = 37) working in the field of maternal health, human rights and health systems identified through personal contacts and references.

### Study selection

We organized the articles from these various sources and search strategies in an excel database. Two people (AG and CB) independently reviewed the titles and abstracts in the database and applied the inclusion and exclusion criteria. Articles on which there was a consensus were included automatically. Articles for which there was a disagreement in application of study criteria were discussed collectively with a third person (AP) before a decision was finalized.

### Data collection process and data items

Data was abstracted by two persons (AG and CB) and regularly discussed among all study authors. Key variables extracted included descriptions of the intervention, target populations, rights awareness promotion elements, facilitators and barriers to implementation, study design, care seeking outcomes (use of antenatal care, birth with a skilled attendant, birth at a facility, skilled care for obstetric complications, postnatal visits, satisfaction with childbirth experience), health outcomes (maternal morbidity, mortality, mental health and prolonged/obstructed labor), human rights outcomes (availability, acceptability, accessibility, quality of care, capacity of rights-holders and bearers) and other relevant outcomes (changes in guidelines, protocols, policies or legislation, stakeholders’ preferences, potential harms, cost).

### Analysis

We did not consider meta-analysis appropriate for these data, as the studies describe a range of interventions implemented over different durations, scale and contexts, with different designs to assess the impact. We therefore conducted a narrative synthesis of the outcomes. In addition, the quality of the studies was independently assessed by two reviewers (AG and CB), using the Effective Public Health Practice Project (EPHPP) quality assessment tool [19].

## Results

### Study selection

**Fig 2:**
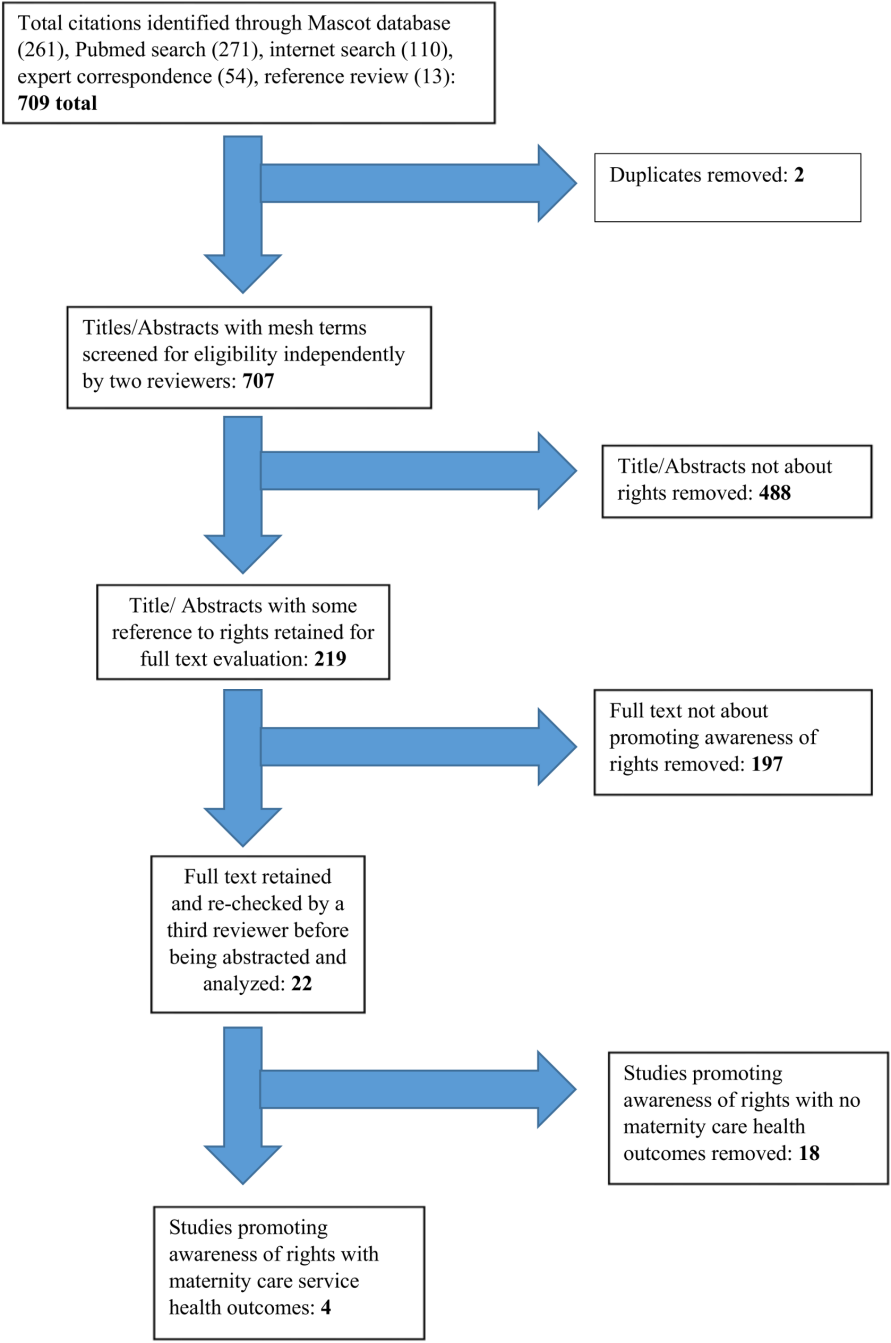
Flow chart outlining study selection results.

The total number of documents in our database from the various sources was 709, with only two duplicates. From the remaining 707 unique documents, 219 made reference to rights in some way. While 22 items discussed promoting awareness of rights for maternal health, only four documented outcomes relevant to this review (Fig 2, S1 File).

### Study characteristics

#### Nature of interventions

The four included studies documented varied interventions, however, all intended to raise awareness of the right to high quality maternity care services. In this section, we provide further details about the nature of each intervention. Table 3 summarizes the scale, duration, study design, quality and study outcomes of each study,

**Table 3:** Studies that promote awareness of rights and document effects on maternal health care-seeking.

Author(s) and Year	Geographic area	Intervention(s)	Study type and quality	Study outcomes ANC	Childbirth
Pandey, Sehfal, Roboud, Levine & Goyal 2007	- Rural India, Uttar Pradesh state-Study in 105 village clusters across 21 districts -Intervention with 22,495 households in 55 village clusters across 11 districts	-4-6 public meetings during two visits spaced two weeks apart to disseminate information about entitled health and education services and village governance -Control areas received no intervention disseminating awareness of rights -Endline surveys carried out one year after the intervention and qualitative data collected two years after the intervention	-Cluster randomized trial -Strong quality	-Multivariate random-effects regression increased prenatal examinations 30%, p<0.001	n/a
Bjorkman & Svensson2009	-Rural Uganda, 4 regions, 9 districts-Study included 50 communities -Intervention with approximately 55,000 households in 25 communities	-A community score card process with a week of meetings when communities and health facility staff review local priorities and action plans and agree on contracts monitored by communities, revisited in meetings six months later -Control areas had no participatory community scorecard intervention -Endline surveys carried out one year after the intervention started	-Cluster randomized trial -Moderate quality	-Difference in difference estimate for the average number of ANC visits provided at facility per month: 5.5 (not significant)	-Difference-in-difference estimates for average facility deliveries per month:-Cross-sectional data: 3.5 (significant at 10%) -Value-added model: 6.3 (significant at 10%)
Ganju, Khanna, Taparia & Hardikar 2014	-Rural India, Dabhva and Sevaniya blocks in Dahod district, Gujarat state-Intervention with 10,374 people in 12 villages	-Over two years local volunteers visit families and prospectively fill a monitoring tool for every woman once during pregnancy and once during post-partum. A report card is developed to dialogue with different stakeholders and support local action.-No control groups	-Participatory action research-Weak quality	-Registration of women for ANC within three months of pregnancy increased from 31.4% to 54.3% in Dhabva block and 17% to 41.8% in Sevaniya block.	-Institutional deliveries increased from 57.1% to 84.6% in Dhabva block and 45.0% to 66.6% in Sevaniya block.
Sinha 2008	-Rural India, Andhra Pradesh state-Intervention with approximately 40,000 people in 37 villages and poor area of headquarter village in 1 district	-Over 15 months awareness raising and community support for pregnant women through local government and youth committees; involvement of their families through monthly meetings; and home visits by a community organizer who worked with families to create a birth preparedness plan and support access to care. -No control groups	-Pre- and post-intervention design-Weak quality	-95.8% of women received 1 ANC Visit (vs. 90.3% at baseline) (p< = 0.001) -72.5% of women received 3+ ANC visits (vs. 61.0% at baseline) (p< = 0.001)	-38.4% delivered at home (vs. 54.1% at baseline) (p< = 0.001)

**Pandey et al. (2007)** focused on spreading awareness about entitled health services through public meetings. Each village was visited twice, with a two-week gap in between. Four to six meetings were held in total, with meetings for low vs. middle and high caste communities held separately. About 250 people or at least 11% of the average population participated in each visit. Each meeting lasted about an hour, during which a 15-minute audiotaped presentation was played, posters and leaflets were distributed and an opportunity to respond to questions with answers prepared ahead of time regarding entitlements to services as per government guidelines (specific days and hours a nurse midwife is available in the village; the obligation of the nurse midwife to provide prenatal and postnatal care; health center availability for more specialized care; where to complain about quality or quantity of health services) was provided.

**Björkman and Svensson (2009)** evaluated a scorecard process that included three types of meetings over five days: (i) a community meeting–over two afternoons with more than 150 community members per day from all spectra of society; (ii) a health facility meeting–a half-day event at the health facility with all staff attending; and (iii) an interface meeting–a half-day event with representatives from the community and the staff attending. In the community meetings, using various participatory methods, the community developed actions plans that they could monitor, suggesting improvements without additional resources for their priority concerns. In the health facility meeting, staff met to discuss how their information on service provision compared to results from household surveys. In the interface meeting, representatives from the community and health facility staff discussed and agreed on a community contract that outlined what needs to be done, and how, when and by whom. After six months, community based organizations (CBOs) facilitated one afternoon community meeting and one afternoon interface meeting to track implementation of the community contract, jointly discuss how to sustain or improve progress or why no progress was made.

**Ganju et al. (2014)** also followed a report card process to raise awareness of women’s rights with regards to safe deliveries. A monitoring tool was developed by combining women’s and medical perspectives of what a safe delivery is, along with government standards regarding maternal health entitlements. Trained volunteers filled out this monitoring tool twice for every pregnant woman through household visits during their eighth month of pregnancy and within 20 days after delivery, which also led to family discussions about women’s rights. Four report cards were generated that tracked progress by colors (red for poor, yellow for average, green for good). Information was shared and discussed first at community level with women’s groups and then with health system authorities and local elected representatives, leading to local action plans that focused on resolving identified problems.

**Sinha (2004)** documented a multi-prong, multi-level initiative to empower communities to make pregnancy safer. At the community level, the project created an environment that respects women and their needs, particularly during pregnancy, through awareness campaigns about rights (posters, street theater, etc.) and the establishment of health and youth committees. Local elected leaders "Gram Panchayat" held regular review meetings with local community and health system stakeholders to resolve issues or raise them at district- and block-level meetings. At the household and individual level, monthly group meetings were held separately with pregnant women, mother-in-laws and husbands, with local health workers. In addition, community organizers followed up with each pregnant woman to create a birth preparedness plan and ensure access to maternity care services as planned.

#### Study quality

According to the (EPHPP) quality assessment tool (EPHPP, 1988), the included studies ranged from strong (Pandey 2007) to moderate (Bjorkman & Svensson 2009) to weak (Ganju et al., 2014, Sinha, 2008). While the studies presented mainly quantitative data, the qualitative data presented was also of low quality.

### Synthesis of results

#### Care-seeking outcomes

As mentioned earlier, findings on health outcomes are detailed in Table 3. With regards to use of ANC, all four studies reported increased use, although in Uganda the increase was not statistically significant and in Uttar Pradesh, India, the differences for low-caste households were also not statistically significant. Three studies reported increases in childbirth in facilities with consistent increases in Uganda, Gujarat, India, and Andhra Pradesh, India.

No studies documented outcomes with regard to birth with a skilled attendant, postpartum visits or skilled care for complications during the early post-partum period, nor impact on health outcomes such as maternal morbidity, mortality, mental health or prolonged/obstructed labor.

#### Human rights outcomes

As this review focused on interventions to raise awareness of rights, in addition to effects on health outcomes, we detail how the studies reported effects on human rights outcomes, such as the availability, acceptability, accessibility and quality of care and the capacity of rights holders and duty bearers.

#### Availability, Acceptability, Accessibility and Quality of Care

In Uttar Pradesh, India, there were no changes in terms of visits by nurse midwives or in development work, although some focus group discussion (FGD) participants noted an improvement in service delivery [20].

In Uganda, absence rates of health facility workers were reported as 13% lower in intervention vs. control facilities and the conditions of primary health unit floors, walls, furniture and smell improved significantly. In addition, waiting times were less for intervention facilities and the use of waiting cards had a significant effect on study outcomes. A difference in difference analysis also indicated a 20% increase in use of equipment during the last health consultation in intervention facilities [21].

In Gujarat, India, weekly antenatal outreach visits, which had been previously suspended at the primary health centers in intervention areas, started within a month of the first report card meeting. Overall regularity and quality of outreach services improved as documented by NGO monitoring tools [22].

In Andhra Pradesh, India, 63.1% of pregnant women reported that auxiliary nurse midwives (ANM) were more regular and responsive, 56.9% reported that the doctor or nurse was more likely to be available at all times in the primary health center, and 53.1% reported that quality of care provided at the primary health center improved, although baseline data is not available for these indicators. In terms of accessibility of care, arrangements for transport prior to birth increased from 28.0% to 52.0% [23].

#### Capacity of rights holders

In Uttar Pradesh, India, while some FGD participants reported that they had raised issues with individual providers, only a few reported doing so in village health council meetings. Some FGD participants also discussed information with others, while those who did not, did not do so because 58.0% believed it was futile, 20.0% did not understand the information well enough and 9.0% were scared [20].

In Uganda, the authors state that the demand-driven mechanism (i.e. community monitoring) was more important than the supply-driven mechanism (provider self-assessment). The authors also indicate that the monitoring tools used by community members and their awareness of health management committee roles and responsibilities were important factors that explain the study outcomes. At the same time, posters on patient’s rights and obligations were not found to be important [21]

In Andhra Pradesh, India, pregnant women reported decreases in their workloads during pregnancy (from 71.7% to 59.7% carrying heavy water, from 52.7% to 40.5% fetching water, from 46.1% to 42.8% engaged in agriculture, from 25.9% to 18.9% for washing clothes and from 30.7% to 6.0% for non-household work) and increases in birth preparedness (from 33.5% to 65.3% discussing birth preparedness plans with family members, 35.5% to 44.5% identifying a birth attendant, 40.2% to 65.3% identifying a hospital/facility for delivery, 30.5% to 49.5% identifying a hospital to go to in an emergency situation, 28.0% to 52.0% making arrangements for transport prior to birth, 43.3% to 67.7% saving money to meet childbirth expenses and 67.1% to 78.6% deciding on a facility delivery) [23].

Study participants in Andhra Pradesh, India, perceived substantive changes at community level and in terms of health care seeking. One medical officer reported the following:

*There has been a lot of change in the last two years*. *A larger number of pregnant women are now visiting the primary health center*. *Moreover*, *they are also following medical advice properly*. *Earlier*, *the situation was totally different*. *If women had a problem*, *they would go to traditional healers/ quacks or use herbal medicine*. *But now all the women take medicines*. *To some extent this change is due to the efforts of community organizers*. *This change was possible because community workers went to the people and made them aware of their rights*. (Medical officer, primary health center, female, 30 years) [23], p.29

#### Capacity of duty bearers

In Uttar Pradesh, India, village council meetings occurred 21.0% more in intervention areas (p = 0.01), however, this was not perceived by FGD participants, as less than 8.0% of them reported that there was any change in the functioning of village council meetings [20].

In Uganda, while health worker knowledge about patient’s rights remained low, it was higher in intervention facilities, and the authors report that the impact of discussing staff performance in local council meetings was related to study outcomes, as was the use of suggestion boxes and posting information on free services in health facilities. Health committees that were not functional were reconstituted in intervention areas and this was reported by study authors as explaining their improved performance [21].

In Gujarat, India, women’s group members mobilized women to attend Mamta Divas (village health and nutrition day for mothers and children) and access services. Community leaders, along with NGO facilitators, became actively involved in tracking the results of the monitoring tools. PHC and block health officers actively engaged in improving quality of care [22].

In Andhra Pradesh, India, pregnant women reported changes in support received from family members during pregnancy and childbirth. While they reported only a 3.0% increase in being accompanied to an antenatal care visit, there was an 11.4% increase in husbands supporting access to health services, 11.2% in providing emotional support and 14.3% in supporting housework. At the community level, while no baseline indicators were collected, at endline 55.7% women reported that more people in the village are talking about maternal health, 64.3% agreed that discussions of safe-delivery increased within the community, 52.9% agreed that community members are more concerned about the safety of pregnant women and 47.9% agreed that community is more committed to improving maternal health [23].

While only 22.8% of women believed that the Gram Panchayat is more active in supporting women during pregnancy/childbirth, review meetings by the Gram Panchayat were reported to have continued by study authors [23]. Local youth and health committees continued to support local volunteer work, generate local funds, support problem solving, and carry out advocacy with higher officials. The NGO that supported the intervention also gained legitimacy and recognition as it was invited by district officials to participate in health meetings. The NGO was encouraged to move from its previous child rights focus to doing more on maternal health and is reported by study authors to be replicating the intervention in other areas.

#### Costs

Pandey et al. (2007) reported that the total cost of disseminating information in a highly scripted manner being USD$4000, which amounts to about USD $0.22 per household in a village cluster of approximately 2500 households in Uttar Pradesh, India. Björkman and Svensson (2009) did not present costs with respect to the maternal health outcomes, but estimated that the cost of averting the death of a child under five is around USD $300 versus USD $887 when using a combined and integrated delivery of 23 interventions shown to reduce mortality from the major causes of death in children younger than five years of age [24].

In Gujarat, India, not a single pregnant woman spent money on childbirth in the third report card in contrast to 62.5% of those who had done so in the first report card in Dhabva block. For Sevaniya block, expenses incurred by women at government hospitals reduced from 55.5% to 25.0% [22].

#### Other study outcomes

Studies did not discuss if there was any effect on broader health system outcomes with regards to changes in guidelines, protocols, policies or legislation.

## Discussion

### Summary of evidence

All of the studies reported improvements in use of antenatal care and in birth in a facility. Of the two higher quality studies, Pandey et al. (2007) detailed improvements in ANC use that were significant; in Björkman and Svensson (2009) these were not significant. For facility births, Björkman and Svensson (2009) documented a significant improvement, while Pandey et al. (2007) did not measure this health outcome.

With regards to human rights outcomes, in terms of availability, acceptability, accessibility and quality of care, Pandey et al. (2007) detailed improvements in perceptions of care, although the study found no improvements to have happened. In contrast, all other studies detailed numerous improvements in availability of health workers, cleanliness of facilities, promptness of treatment and accessibility of care.

In terms of capacity of rights holders, Pandey et al. (2007) found that while respondents did discuss information with others, significant barriers remained to acting on such information. In Uganda, posters on patient’s rights and obligations were not found to have significant effects, in contrast to community monitoring and awareness of health management committee roles [21]. In Andhra Pradesh, India, pregnant women were reported to be more aware of their health, entitlements [22] [23] and reported being able to decrease their workloads during pregnancy and save money in anticipation of delivery costs [23].

With regards to the capacity of duty bearers, in Uttar Pradesh, India, while village council meetings did occur more frequently in intervention areas, this was not perceived to be the case by respondents [20] In Uganda, health worker knowledge of patient’s rights remained low but increased and numerous mechanisms to report on their performance led to improved health outcomes. In Gujarat, India, and Andhra Pradesh, India, multiple stakeholders, whether family members, women’s groups, community leaders and local committees, or health workers all got involved in improving maternal health [22] [23].

Costs reported in Uttar Pradesh,India, and Uganda seemed to suggest that the interventions were not expensive to implement [20] [21] and in Gujarat, India, project monitoring tools reported that costs of accessing services declined for women [22].

There were no changes in broader health system outcomes with regards to changes in guidelines, protocols, policies or legislation reported by any of the studies. With regards to potential or actual harms or risks very little was documented or discussed.

### Limitations

Strengths of this review include the concerted effort to search multiple sources of information, to overcome the challenge of the paucity of documentation of interventions that promote awareness of rights for maternal and newborn health with information on health outcomes. Even with an extensive search through databases and search engines, three of the four studies included in the review were identified through expert consultation. These were not categorized by databases as promoting awareness of rights, as they do not mention this in their title, abstract or key words, even though they explicitly mention promoting awareness of rights as part of their intervention.

Of the 22 studies identified, many reported that they promoted awareness of rights, but then failed to elaborate how they did so at all or failed to do so with sufficient detail. For example, many projects supported a rights-based ethos (supporting women’s rights to privacy and decision-making, male engagement, respectful care) without specifying how they operationalized such measures and clarifying whether they explicitly promoted awareness of rights alongside such efforts.

Of those studies that did detail initiatives that promoted awareness of rights, very few systematically documented linkages to health care seeking, benefits and harms to target populations, values and preferences of stakeholders engaged, or cost implications for programs or households. Sinha (2008) mentioned there was some initial resistance to raising issues of women’s rights particularly with men, but that these were overcome by project members through further discussion and the strategic use of humor. Considering that interventions that support promoting the awareness of rights are meant to empower individuals to change the status quo or challenge power relations, the lack of information on conflict or backlash is particularly striking.

Three of the four studies were conducted in India and all of them were in rural communities, which further limits our ability to generalize the findings.

## Conclusions

Our review compliments findings from other reviews that have sought to establish the general impact of human rights on maternal and newborn health [16]. We focused particularly on efforts to promote awareness of rights, just as Ferguson & Halliday (2013) focused on community participation as one way of implementing rights-based approaches, and faced similar challenges in doing so. Our findings reflect the nature of health and human rights literature in that it mainly consists of social analysis with epidemiological studies being a minority [25], partly explained by the normative disciplinary foundation of human rights work, in contrast to the empirical basis for public health evidence [26]. While the basis for supporting a rights-based approach draws on normative guidance agreed upon by States and the United Nations, the programmatic evidence base from which to guide public health implementation remains weak and needs strengthening.

Our review followed standard public health practice for systematic reviews and used specific criteria focused on examining effects on increasing use of maternity care services. Given the lack of studies that measured health effects, future reviews should examine the larger body of literature that exists on promotion of rights for other sexual and reproductive health issues.

Based on this review, the effect of promoting awareness of rights on improved maternal health outcomes is limited to rural populations primarily in India and Uganda. Studies from other regions of the world, in different settings (e.g. urban) and with varied populations that may face particular forms of discrimination and exclusion (migrants, nomadic groups, young people, institutionalized populations, disabled, etc.) are needed.

As well as further research, explicit promotion of awareness of rights in current health programming is warranted. Promoting awareness of rights is one element of increasing demand for and use of quality maternity care services for women during pregnancy, birth and after birth. Unless programs are not using the language of rights for strategic reasons, many projects that build individual and community capacity to improve demand for and access to quality maternity care services lend themselves to complimentary actions to promote awareness of rights. However, they cannot be evaluated as doing so, if they don’t mention this or explain how they incorporated such an approach into their work. Existing studies that implement rights-based approaches need to be better labelled so that they can be more easily identified; and further studies of robust design to measure the contribution of interventions that promote awareness of rights as one important element of increasing access and use of quality skilled care for women during pregnancy, birth and after birth are required.

## Supporting Information

S1 File PRISMA checklist. (DOC)
